# Advances in multi-modal non-invasive imaging techniques in the diagnosis and treatment of polypoidal choroidal vasculopathy

**DOI:** 10.3389/fmed.2023.1221846

**Published:** 2023-07-27

**Authors:** Yuelin Wang, Xingwang Gu, Youxin Chen

**Affiliations:** ^1^Department of Ophthalmology, Peking Union Medical College Hospital, Chinese Academy of Medical Sciences, Beijing, China; ^2^Key Laboratory of Ocular Fundus Diseases, Chinese Academy of Medical Sciences, Beijing, China

**Keywords:** polypoidal choroidal vasculopathy (PCV), multimodal, non-invasive imaging technology, diagnosis, treatment

## Abstract

Polypoidal choroidal vasculopathy (PCV) is a disease characterized by subretinal pigment epithelium (RPE) orange-red polypoidal lesions and abnormal branching neovascular networks (BNNs). In recent years, various non-invasive imaging technologies have rapidly developed, especially the emergence of optical coherence tomography angiography (OCTA), multi-spectral imaging, and other technologies, which enable the observation of more features of PCV. In addition, these technologies are faster and less invasive compared to indocyanine green angiography (ICGA). Multi-modal imaging, which combined multiple imaging techniques, provides important references for the diagnosis and treatment of PCV with the assistance of regression models, deep learning, and other algorithms. In this study, we reviewed the non-invasive imaging techniques, multi-modal imaging diagnosis, and multi-scene therapeutic applications of PCV, with the aim of providing a reference for non-invasive multi-modal diagnosis and treatment of PCV.

## 1. Introduction

Polypoidal choroidal vasculopathy (PCV) is a disease characterized by orange-red polypoidal lesions (PLs) and abnormal branching neovascular networks (BNNs) (1–3). It was first described by Yannuzzi in 1982 and was officially published in 1990 (4). Compared to wet age-related macular degeneration (wAMD), PCV has the characteristics of recurrent hemorrhage, subretinal hemorrhage, lower age of onset, and poor response to anti-VEGF therapies. The mechanism of PCV is still unclear, and it is a difficult and hot topic in the field of retinal research due to its serious harm (5, 6).

The gold standard for the diagnosis of PCV in the past was indocyanine green angiography (ICGA) (7). This involves observing focal hyperfluorescent lesions within 6 min on ICGA, as well as at least one of the following: observation of BNN on ICGA, visible pulsation on dynamic ICGA, nodular appearance when ICGA viewed stereoscopically, hypofluorescent halo on ICGA, orange subretinal nodule on a color photograph, and massive subretinal hemorrhage. This examination requires venipuncture and the injection of contrast agents into the body, which carry risks such as systemic allergies and local infections. Therefore, it is a contradiction for some patients. In remote areas, many PCV patients have not been correctly diagnosed due to the lack of ICGA examination equipment.

In recent years, non-invasive imaging techniques have been rapidly developed. Some non-invasive imaging techniques, such as optical coherence tomography (OCT), optical coherence tomography angiography (OCTA), and multi-spectral imaging, have emerged through the limitations of traditional non-invasive imaging techniques (fundus photography, autofluorescence, etc.) and provided more observational dimensions for the diagnosis of PCV. In addition, with the advent of multi-modal imaging technology and the emergence of artificial intelligence algorithms, the diagnosis and treatment of PCV have gradually become less dependent on invasive ICGA examinations.

Therefore, we review recent literature and summarize the latest advances in imaging features, multi-modal diagnosis, and multi-scenario treatment applications of PCV under non-invasive imaging techniques.

## 2. Non-invasive imaging PCV techniques

### 2.1. Fundus photography

Fundus photography can be taken using a traditional camera to capture the patient's dilated fundus or using a non-dilated camera to record the characteristics of PCV in the fundus posterior pole (8). The polypoidal lesion of PCV (formerly known as a polyp) can appear as one or more orange-red nodules under the retina, which can occur in the locations of the macular area, near the optic disk, near the vascular arcade, and even in the mid-periphery, but most of the polypoidal lesions appear in the posterior pole. In addition to the polypoidal lesions, extensive subretinal hemorrhage is also an important feature of some PCV cases, with a range often >4 disk diameters (DDs), located under the retina or RPE, and sometimes combined with vitreous hemorrhage (9).

### 2.2. Autofluorescence

Fundus autofluorescence (FAF) is a non-invasive diagnostic technique based on the theory that pigments and molecules can emit autofluorescence when excited by light. FAF can provide information about RPE and photoreceptor cell metabolism and activity. Oztas et al. (10) found that PLs may appear as granular low autofluorescence. Zhao et al. (11) found that FAF features of PLs include 49.8% high fluorescence rings, 22.6% mixed fluorescence with low fluorescence, and 3.7% mixed fluorescence with polyps, but there are still some (8.2%) PCV polyps with no obvious FAF changes. Some studies have also reported that autofluorescence can observe more pachydrusen, accounting for 61.8% of PCV (12). Overall, FAF has lower diagnostic efficacy for PCV.

### 2.3. OCT

Optical coherence tomography (OCT) is a non-invasive imaging technique that uses light waves to capture detailed images of the retina and other structures in the eye (13). Initially, the scanning rate of OCT was only a few hundred times per second, and the imaging quality was low. However, recent OCT technology can achieve scanning speeds of up to 400,000 times per second, a scanning width of 24 mm, and a scanning depth of 6 mm, bringing new momentum to clinical research (14, 15).

In earlier years, time-domain OCT technology was able to roughly display the inverted V-shaped pigment epithelium detachment (PED) of PCV, but the detailed features of the polyps could not be shown (16). Swept-source OCT (SS-OCT) was introduced in 2012, which used a longer wavelength light source and a tunable laser to sweep through a range of frequencies, producing a high-resolution image of the eye.

Some retinal and choroidal measurements obtained from SS-OCT are important parameters for quantifying the anatomical structure of the PCV. The central macular thickness (CMT) refers to the distance between the inner limiting membrane (ILM) and the inner boundary of the RPE (17). Generally, active polyps may cause retinal edema and hemorrhage, leading to an increase in CMT values and affecting the patient's vision. After anti-VEGF treatment, the CMT of some patients may decrease, and thus monitoring the CMT can be used to evaluate treatment efficacy (18). The subfoveal choroidal thickness (SFCT) refers to the distance between the Bruch's membrane and the choroid–scleral interface of the central fovea of the macula. Currently, PCV can be classified into type 1 and type 2 based on SFCT and other parameters, and there are reports suggesting that type 2 PCV have a different genotype, thicker SFCT, and poor responsiveness to anti-VEGF therapy as compared with type 1 PCV (19, 20).

In 2022, the PCV working group of the Asia-Pacific Academy of Ophthalmology published the main OCT features of PCV in the journal Ophthalmology (21), which include the following: (1) Sub-RPE ring-like lesion, also known as bubble sign, refers to a round structure observed under PED and characterized by hyporeflective center and a hyperreflective outline; (2) Complex or multi-lobular PED, which is characterized by notches on the PED, resembling an “M” shape with visible multi-lobular PED; (3) Sharp peak PED, also known as a thumb-like protrusion, presents as a narrow-peaked PED with an inverted “V” configuration, with a peak angle of >70 degrees on at least one side and a base-to-width ratio of >1; (4) A double-layer sign refers to the separation of the undulating RPE line and the underlying Bruch's membrane, forming a double layer; (5) Thick choroid with dilated Haller's layer vessels, which are characterized by a choroidal thickness of ≥300 μm at the foveal center, Haller's layer vessel dilation occupying most of the choroidal thickness, and thinning of the choroidal capillaries above it; (6) Complex or multi-lobular PED is visible as multiple high-reflective branching vascular networks of PED on en face OCT; (7) Fluid compartment is characterized by obvious subretinal fluid, which may be accompanied by intraretinal fluid.

### 2.4. OCTA

The OCTA technology obtains three-dimensional data images by establishing an en face mode based on the traditional cross-sectional scan (B-scan) combined with a coronal scan (C-scan). By identifying the displacement changes in blood flow signals, it can construct the morphology and structure of retinal vessels, choroidal blood flow, and neovascularization (22, 23).

In the past few years, SD-OCTA has provided important information on PLs and BNNs in PCV. For PLs, SD-OCTA showed that these lesions are often located between Bruch's membrane and the RPE layer. The lesion morphology is often “ring-shaped”, but it can also be seen as “cluster-like”, “dot-like”, “ring-like”, or “nodular”. However, due to the variability of blood flow signals and the limitations of SD-OCTA scanning speed, the detection rate of PLs by SD-OCTA is approximately 17.0 to 92.3% (24). For BNNs, Huang et al. (25) proposed three types of BNN morphology, namely, “Trunk” pattern, “Glomeruli” pattern, and “Stick” pattern, with detection rates of approximately 70.0 to 100.0% and sensitivities of 0.67 and 0.86. Wang et al. (26) conducted a meta-analysis and discovered that the average detection rate of BNN (0.86) in PCV was higher compared to the detection rate of PLs (0.67) in studies primarily utilizing SD-OCTA as the detection tool. This difference in detection rates may be attributed to the faster blood flow velocity observed in BNN compared to that of the polypoidal lesions in PCV. Meanwhile, OCTA combined with enhanced depth imaging (EDI) technology can provide more information on PCV. The choroidal vascularity index (CVI) is defined as the ratio between vascular luminal area (LA) and total choroidal area (TCA), which is the sum of LA and choroidal stromal area (SA) on EDI-OCT. Batioglu et al. observed that typical PCV (a subtype of PCV) has a higher choroidal blood flow and CVI compared to polypoidal CNV (17). In addition, intervortex venous anastomoses were observed in PCV, and more dilated anastomotic vessels were observed in the typical PCV (17).

In recent years, SS-OCTA has been developed with advantages such as faster scanning speed and deeper scanning depth, providing new insights into the understanding of PCV. Bo et al. (27) analyzed 23 eyes diagnosed with PCV by ICGA and found that SS-OCTA was able to detect all 43 polypoidal lesions identified by ICGA, displaying them as tangled vessels. In addition, SS-OCTA detected 16 tangled vascular structures that were not observed on ICGA. Similarly, BNNs were detected in all eyes on SS-OCTA, while ICGA only identified BNNs in 17 eyes (74%). Kim et al. (28) also conducted a comparative study of ICGA and SS-OCTA in the diagnosis of PCV. They found that SS-OCTA could identify a larger number of polypoidal lesions than ICGA in 12 eyes and detect BNNs in all cases. There was no significant difference in the measurement of the PCV lesion area between ICGA and SS-OCTA images. These results suggest that SS-OCTA imaging is comparable to, and may even be superior to, ICGA for the detection of PLs and BNNs.

### 2.5. Multi-spectral imaging

Multi-spectrum imaging (MSI) is an imaging method that records images in spectral form and has been attempted for the diagnosis of PCV. Ma et al. (29) found that PLs can be observed at wavelengths of 680 nm and above as lobes of “hyperborder, hypocenter” pattern lesions, and BNN can be vaguely observed as an area of interlacing hyper-reflectance with linear or dot of signals. Subretinal fluid appears as local blurred hyperreflective areas under short wavelengths, and PED can appear as a slightly high-reflective border. The sensitivity and specificity of MSI for diagnosing PCV are 0.84 and 0.93, respectively, with positive and negative predictive values of 0.94 and 0.81, respectively. Therefore, multi-spectrum imaging has a certain diagnostic value for PCV.

## 3. Multi-modal non-invasive imaging diagnosis

### 3.1. Theoretical basis

Multi-modal imaging is a joint imaging diagnostic technique. It combines multiple imaging modalities, complementing each other's strengths, and achieves rapid and accurate diagnosis of complex and difficult fundus diseases through mutual “verification”. For example, in the field of tumor radiology, integrating multiple modal magnetic resonance sequences can improve diagnostic efficiency. On the one hand, the data from multiple modalities come from different imaging devices, with large amounts of data and more comprehensive descriptions of diseases. On the other hand, in regression analysis, or even deep-learning analysis, the “interaction” between modalities can increase the amount of diagnostic information, especially in deep learning, where the connection layer between each vector connects the information, making the diagnostic ability more advantageous than single-modal imaging.

### 3.2. Combined diagnosis of PCV

Multi-modal diagnosis of PCV has various combinations, mainly including OCT combined with fundus photography and OCT combined with OCTA (8, 21, 30–36) (Table 1).

**Table 1:** Diagnostic accuracy of multi-modal non-invasive imaging techniques for the diagnosis of PCV.

**References**	**Non-invasive diagnostic combination**	**Diagnostic criteria**	**Meeting criteria**	**Sensitivity**	**Specificity**	**PPV**	**NPV**	**AUC**
Chaikitmongkol et al. (30)	FP+OCT	Four diagnostic criteria: (1) notched or hemorrhagic PED on fundus photography; (2) sharply peaked PED detected on OCT; (3) notched or multi-lobulated PED on OCT; and (4) hyperreflective ring underneath PED detected on OCT.	≥1 criterion	0.98	0.67	0.76	0.98	0.83
			≥2 criteria	0.95	0.95	0.92	0.95	0.93
			≥3 criteria	0.84	0.95	0.95	0.85	0.90
			4 criteria	0.53	0.97	0.94	0.66	0.75
Yang et al. (31)	FP+OCT	Five diagnostic criteria: (1) subretinal orange nodule on FP; (2) thumb-like PED on OCT; (3) notched PED on OCT; (4) bubble sign on OCT; and (5) Bruch's membrane depression under serosanguinous PED on OCT.	≥1 criterion	0.92	0.75	0.79	0.90	0.83
			≥2 criteria	0.88	0.92	0.92	0.89	0.90
			≥3 criteria	0.81	0.96	0.95	0.83	0.88
			≥4 criteria	0.69	0.98	0.97	0.76	0.84
			5 criteria	0.46	1.00	1.00	0.65	0.73
Chaikitmongkol et al. (8)	FP+OCT	(1) Fundus photograph indications: orange-red nodules, or hemorrhagic or fibrovascular PED, or extensive subretinal hemorrhage, and other characteristics; (2) OCT indications: sharp PED peak, or PED notch, or circular lesion below RPE, or double-layer sign, and other features.	Meet both indications	0.83	0.83	0.77	0.88	0.83
Chong Teo et al. (32)	FP+OCT	Three diagnostic criteria: (1) sharp-peaked PED on OCT, (2) sub-RPE ring-like lesion on OCT, and (3) orange nodule on FP.	Meeting all criteria	0.65	0.82	0.68	0.88	0.85
Cheung et al. (21)	FP+OCT	Three major criteria: (1) sub-RPE ring-like lesion on OCT; (2) sharp-peaked PED on OCT; and (3) en face OCT complex RPE elevation. Four minor criteria: (1) orange nodule on FP; (2) complex or multi-lobular PED on OCT; (3) thick choroid with dilated Haller's layer on OCT; and (4) double-layer sign on OCT.	Meet three major criteria and one minor criterion	0.78	0.91	0.94	0.68	0.91
Cheung et al. (33)	OCT+OCTA	Three OCT criteria: (1) notched/narrow-peaked PED; or (2) round sub-RPE hyporeflective lesion, or (3) any RPE detachment. Two OCTA criteria: (1) localized sub-RPE hyperflow lesion in the cross-sectional OCTA; or (2) nodular hyperflow lesion in the en face OCTA.	Meet both OCT and OCTA criteria.	0.82	1.00	1.00	0.87	—
de Carlo et al. (34)	OCT+OCTA	(1) OCT criteria: PCV complex with small highly reflective circles representing polyps adjacent to large highly reflective circles in a “snowman” sign or adjacent to geographically shaped BNNs, etc. (2) OCTA criteria: the presence of an inverted U shape (the polyp) next to a large serous PED or shallow elevation of the RPE above Bruch membrane (“double layer” sign), etc.	Meet both OCT and OCTA criteria.	0.44	0.87	—	—	—
Chou et al. (35)	FP+OCT+AI	—	—	0.81	0.85	—	—	0.84
Xu et al. (36)	FP+OCT+AI	—	—	0.89	0.96	—	—	0.87

AI, artificial intelligence; AUC, area under the curve; BNN, branching neovascular network; FP, false positive; NPV, negative predictive value; OCT, optical coherence tomography; OCTA, optical coherence tomography angiography; PED, pigment epithelial detachment; PPV, positive predictive value; RPE, retinal pigment epithelium.

In a joint multi-modal study of fundus photography and OCT, Yang et al. (31) found that by incorporating features such as subretinal orange-red nodules on fundus photography, thumb-like PED, notched PED, bubble sign, and Bruch's membrane depression under serosanguinous PED on OCT, the diagnostic strategy using at least two of the five features had a sensitivity of 0.88 and specificity of 0.92 for diagnosing PCV. Chaikitmongkol et al. (30) observed that the presence of at least two of the four features (notched or hemorrhagic PED detected using color fundus photography, sharply peaked PED detected using OCT, notched or multi-lobulated PED detected using OCT, and hyperreflective ring underneath PED detected using OCT) could diagnose PCV with a sensitivity and specificity of over 90%. Using fundus photography and OCT manifestations, the Asia-Pacific Ocular Imaging Society PCV Workgroup found, through regression analysis, that the positive predictive value for diagnosing PCV reached 0.93 when sharp PED peak, sub-RPE ring-like lesion, and En face OCT complex RPE elevation were observed simultaneously. Adding one of the criteria of observation of an orange nodule on fundus photography, complex or multi-lobular PED, thick choroid with dilated Haller's layer vessels, and double-layer sign could increase the positive predictive value to 0.94.

In a multi-modal study combining OCT and OCTA, de Carlo et al. (34) found that the sensitivity and specificity of OCT for diagnosing PCV were 0.30 and 0.86, respectively, while the multi-modal combination of OCT and OCTA improved the sensitivity to 0.44 with a specificity of 0.87. Similarly, Cheung et al. (33) found that the sensitivity for the combined diagnosis of PCV and CNV/AMD using a two-step diagnostic algorithm with OCT and OCTA was still 0.82 when adding OCTA information to the OCT diagnosis, but the specificity increased from 0.52 to 1.00. This suggests that the diagnostic efficiency of multi-modal non-invasive diagnosis of PCV improved compared to single-modality diagnosis.

### 3.3. Artificial intelligence assistant diagnosis

In the field of artificial intelligence, multi-modal machine learning combines the advantages of each modality through algorithms and other techniques. Single modality refers to the use of a single type of data or input modality to train a machine learning model. Multi-modal learning refers to the use of the complementarity between multiple modalities and the interaction between each fully connected layer to learn better features.

In some studies, artificial intelligence has been used in the non-invasive diagnosis of PCV. Wongchaisuwat et al. (37) developed a model that trained on 2,334 OCT images of PCV using ResNet. On the internal dataset, the model achieved a diagnostic sensitivity and specificity of 1.00 and 0.60, respectively, whereas, on the external dataset, the model achieved a sensitivity and specificity of 0.85 and 0.71, respectively. Chou et al. (35) trained a deep-learning model based on color fundus photography and OCT biomarkers. They introduced a multiple correspondence analysis algorithm to train the PCV diagnostic model with a transfer learning architecture on approximately 700 cases, achieving a sensitivity and specificity of 0.81 and 0.85, respectively. Xu et al. (36) developed a bimodal deep-learning convolutional neural network model (DCNN-Combo) based on color fundus photography and OCT that achieved a PCV diagnosis with an accuracy of 87.4%, which was even more consistent with the diagnostic gold standard than that of ophthalmologists.

## 4. Application of multi-modal non-invasive imaging in PCV treatment

### 4.1. Monitoring the treatment response of PCV

Multiple studies have reported the use of non-invasive methods, such as fundus photography, OCT, and OCTA, to evaluate the response to PCV treatment. Cho et al. (38) reported that based on the manifestation of fundus photography, 10% of eyes in PCV patients at 5 years and 30% of eyes at 10 years could observe subretinal hemorrhage in fundus photography, which is significantly higher than typical nAMD, indicating disease progression.

Using SS-OCT/SS-OCTA gives us the ability to measure the choroid in PCV before and after treatment. Tan et al. (39) reported the use of OCT to evaluate the treatment response of PCV patients and assessed whether the polyp was closed by monitoring the changes such as (1) the disappearance of subretinal fluid, (2) a significant reduction of PED height, (3) an increased reflection within PED, (4) the disappearance of low-reflection ring, and (5) a reduction of thickened RPE structure. The diagnosis AUC reached 0.90 when the criteria of (1), (3), and (4) were met. Montorio et al. (40) used OCTA to evaluate changes in choroidal capillary flow density and SFCT and found that the choroidal capillary flow density in PCV treated with anti-VEGF drugs was significantly reduced, and the CMT and SFCT were also significantly decreased. Fukuyama et al. (41) found that the blood flow signal within the polyp on OCTA was related to the response of PCV to anti-VEGF drug treatment during a follow-up of more than 6 months, and a low blood flow signal within the polyp lasting for more than 2 weeks may indicate poor initial treatment response of PCV.

### 4.2. Monitoring of PCV recurrence

Some studies use OCT and fundus photography to monitor PCV recurrence. Bo et al. (42) defined exudative recurrence as the reappearance of IRF, SRF, or serous PED on OCT, or the appearance of new subretinal or RPE hemorrhage on fundus photography. Ma et al. (43) described the impact of different BNN patterns observed on OCTA on the treatment response and outcomes of PCV and found that type 2 (“Glomeruli” pattern) and type 3 (“Stick” pattern) were associated with thicker choroid and increased recurrence of PCV, while type 1 (“Trunk” pattern) was associated with thin choroid, vitelliform lesions, and AMD-like features. However, because OCTA cannot display vascular leakage, it is currently primarily used in combination with structural OCT for treatment monitoring and as a reference for recurrence treatment decisions.

### 4.3. Timing of rescue PDT

The APOIS PCV working group also reported the criteria for identifying PCV in eyes previously diagnosed with wAMD and with poor response to three injections of anti-VEGF treatment (32). When one of the following three criteria is met, supplementary PDT treatment may be considered: orange-red nodules observed on fundus photography, peaked PED observed on OCT, and low-reflectance ring structure observed on OCT. The combination of these three criteria has a specificity of 0.82 and a sensitivity of 0.65 (AUC of 0.85) for detecting PCV.

## 5. Limitations in non-invasive multi-modal imaging diagnosis of PCV

Although non-invasive multi-modal imaging has a certain value in the diagnosis and treatment of PCV, there are still several problems. First, the pathogenesis of PCV is not yet clear, and the disease classification is complex (44). Some scholars believe that the high fluorescence signal observed on ICGA may be a common manifestation of many diseases, so the use of other non-invasive imaging for the identification and diagnosis of PCV may lead to misdiagnosis (45). Second, the current diagnostic criteria for a combined diagnosis are mostly for newly diagnosed PCV. However, many patients are misdiagnosed with other diseases, and the possibility of PCV is only considered after various treatments have been proven ineffective, which may affect the diagnosis of PCV by non-invasive multi-modal imaging due to changes in some PED and intraretinal fluid (46). Third, different non-invasive multi-modal detection instruments may have different parameters and may affect the accuracy of diagnosis, such as image quality rating, degree of ocular refractive media opacity, and instrument resolution (47).

## 6. Conclusion

In summary, non-invasive imaging diagnostic technologies provide fast and safe diagnostic methods with high accuracy and are comparable to or might be better than ICGA in some aspects. The superiority of multi-modal non-invasive imaging diagnostic technology, combined with multiple devices and special algorithms, fully leverages their interactive effects and has played an important role in the diagnosis and treatment of PCV.

## Author contributions

YW and XG were involved in manuscript drafting. YC contributed to the manuscript revision. All authors have read and approved the final manuscript.

## References

[1] Sen P Manayath G Shroff D Salloju V Dhar P. Polypoidal choroidal vasculopathy: an update on diagnosis and treatment. Clin Ophthalmol. (2023) 17:53–70. 10.2147/OPTH.S385827 36636621 PMC9831529

[2] Koh AH Expert PCVP Chen LJ Chen SJ Chen Y Giridhar A. Polypoidal choroidal vasculopathy: evidence-based guidelines for clinical diagnosis and treatment. Retina. (2013) 33:686–716. 10.1097/IAE.0b013e3182852446 23455233

[3] Xue Y Qinhua C. Short-term efficacy in polypoidal choroidal vasculopathy patients treated with intravitreal aflibercept or conbercept. Front Med (Lausanne). (2022) 9:835255. 10.3389/fmed.2022.835255 35252267 PMC8891458

[4] Yannuzzi LA Sorenson J Spaide RF Lipson B. Idiopathic polypoidal choroidal vasculopathy (IPCV). Retina (Philadelphia, Pa). (1990) 10:1–8. 10.1097/00006982-199001010-00001 1693009

[5] Han J Choi S Park JI Hwang JS Han JM Ko J. Detecting macular disease based on optical coherence tomography using a deep convolutional network. J Clin Med. (2023) 12:1005. 10.3390/jcm12031005 36769653 PMC9917826

[6] Ruamviboonsuk P Cheung GCM Chainakul M Arjkongharn N Chan HH Ogura Y. Updates on risk factors, diagnosis, and treatments of polypoidal choroidal vasculopathy. Asia Pac J Ophthalmol. (2023) 12:184–95. 10.1097/APO.0000000000000573 36728294

[7] Koh A Lee WK Chen LJ Chen SJ Hashad Y Kim H. EVEREST study: efficacy and safety of verteporfin photodynamic therapy in combination with ranibizumab or alone versus ranibizumab monotherapy in patients with symptomatic macular polypoidal choroidal vasculopathy. Retina. (2012) 32:1453–64. 10.1097/IAE.0b013e31824f91e8 22426346

[8] Chaikitmongkol V Khunsongkiet P Patikulsila D Ratanasukon M Watanachai N Jumroendararasame C. Color fundus photography, optical coherence tomography, and fluorescein angiography in diagnosing polypoidal choroidal vasculopathy. Am J Ophthalmol. (2018) 192:77–83. 10.1016/j.ajo.2018.05.005 29753852

[9] Li MS Tsen CL. Clinical features and outcomes of breakthrough vitreous hemorrhage secondary to polypoidal choroidal vasculopathy. PLoS ONE. (2022) 17:e0279778. 10.1371/journal.pone.0279778 36584198 PMC9803236

[10] Oztas Z Mentes J Nalcaci S Baris M. Characteristics of fundus autofluorescence in active polypoidal choroidal vasculopathy. Turk J Ophthalmol. (2016) 46:165–8. 10.4274/tjo.54280 28058151 PMC5200821

[11] Zhao X Xia S Chen Y. Characteristic appearances of fundus autofluorescence in treatment-naive and active polypoidal choroidal vasculopathy: a retrospective study of 170 patients. Graefes Arch Clin Exp Ophthalmol. (2018) 256:1101–10. 10.1007/s00417-018-3980-2 29656364

[12] Lai TYY Tang Z Lai ACW Szeto SKH Lai RYK Cheung CY. Association of fundus autofluorescence abnormalities and pachydrusen in central serous chorioretinopathy and polypoidal choroidal vasculopathy. J Clin Med. (2022) 11:5340. 10.3390/jcm11185340 36142987 PMC9500611

[13] Permadi AC Djatikusumo A Adriono GA. Optical coherence tomography in diagnosing polypoidal choroidal vasculopathy. Looking into the future: a systematic review and meta-analysis. Int J Retina Vitreous. (2022) 8:14. 10.1186/s40942-022-00365-5 35227320 PMC8883730

[14] Laíns I Wang JC Cui Y Katz R Vingopoulos F Staurenghi G. Retinal applications of swept source optical coherence tomography (OCT) and optical coherence tomography angiography (OCTA). Prog Retin Eye Res. (2021) 84:100951. 10.1016/j.preteyeres.2021.100951 33516833

[15] Yamashiro K Yanagi Y Koizumi H Matsumoto H Cheung CMG Gomi F. Relationship between pachychoroid and polypoidal choroidal vasculopathy. J Clin Med. (2022) 11:4614. 10.3390/jcm11154614 35956229 PMC9369798

[16] Kumar A Kumawat D Sundar MD Gagrani M Gupta B Roop P. Polypoidal choroidal vasculopathy: a comprehensive clinical update. Ther Adv Ophthalmol. (2019) 11:2515841419831152. 10.1177/2515841419831152 30834360 PMC6393826

[17] Batioglu F Yanik O Ozer F Demirel S Ozmert E. A comparative study of choroidal vascular and structural characteristics of typical polypoidal choroidal vasculopathy and polypoidal choroidal neovascularization: OCTA-based evaluation of intervortex venous anastomosis. Diagnostics (Basel). (2022) 13:138. 10.3390/diagnostics13010138 36611430 PMC9818584

[18] Pant P Kundu A Rathinavelu JK Wei X Agrawal R Stinnett SS. Longitudinal assessment of the choroidal vascularity index in eyes with branch retinal vein occlusion-associated cystoid macular edema. Ophthalmol Ther. (2023) 12:2103–15. 10.1007/s40123-023-00731-y 37221425 PMC10287880

[19] Miki A Honda S Kondo N Negi A. The association of age-related maculopathy susceptibility 2 (ARMS2).and complement factor H (CFH).variants with two angiographic subtypes of polypoidal choroidal vasculopathy. Ophthalmic Genet. (2013) 34:146–50. 10.3109/13816810.2012.749288 23289808

[20] Tanaka K Mori R Kawamura A Nakashizuka H Wakatsuki Y Yuzawa M. Comparison of OCT angiography and indocyanine green angiographic findings with subtypes of polypoidal choroidal vasculopathy. Br J Ophthalmol. (2017) 101:51–5. 10.1136/bjophthalmol-2016-309264 27913447

[21] Cheung CMG Lai TYY Teo K Ruamviboonsuk P Chen SJ Kim JE. Polypoidal choroidal vasculopathy: consensus nomenclature and non-indocyanine green angiograph diagnostic criteria from the asia-pacific ocular imaging society PCV workgroup. Ophthalmology. (2021) 128:443–52. 10.1016/j.ophtha.2020.08.006 32795496

[22] Chen L Yuan M Sun L Chen Y. Different morphology of branching neovascular network in polypoidal choroidal vasculopathy: a swept-source optical coherence tomography angiography study. J Clin Med. (2023) 12:742. 10.3390/jcm12030742 36769390 PMC9918075

[23] Yuan MZ Chen LL Yang JY Luo MY Chen YX. Comparison of OCT and OCTA manifestations among untreated PCV, neovascular AMD, and CSC in Chinese population. Int J Ophthalmol. (2020) 13:93–103. 10.18240/ijo.2020.01.14 31956576 PMC6942943

[24] Li B Chen Y. Application and research progress of optical coherence tomography angiography in polypoidal choroidal vasculopathy. Chinese J Ophthalmol. (2020) 56:950–5. 10.3760/cma.j.cn112142-20200509-00259 33342122

[25] Huang CH Yeh PT Hsieh YT Ho TC Yang CM Yang CH. Characterizing branching vascular network morphology in polypoidal choroidal vasculopathy by optical coherence tomography angiography. Sci Rep. (2019) 9:595. 10.1038/s41598-018-37384-y 30679701 PMC6345899

[26] Wang Y Yang J Li B Yuan M Chen Y. Detection rate and diagnostic value of optical coherence tomography angiography in the diagnosis of polypoidal choroidal vasculopathy: a systematic review and meta-analysis. J Ophthalmol. (2019) 2019:6837601. 10.1155/2019/6837601 31915542 PMC6931027

[27] Bo Q Yan Q Shen M Song M Sun M Yu Y. Appearance of polypoidal lesions in patients with polypoidal choroidal vasculopathy using swept-source optical coherence tomographic angiography. JAMA Ophthalmol. (2019) 137:642–50. 10.1001/jamaophthalmol.2019.0449 30998817 PMC6567849

[28] Kim K Yang J Feuer W Gregori G Kim ES Rosenfeld PJ. A comparison study of polypoidal choroidal vasculopathy imaged with indocyanine green angiography and swept-source optical coherence tomography angiography. Am J Ophthalmol. (2020) 217:240–51. 10.1016/j.ajo.2020.05.017 32445699

[29] Ma F Yuan M Kozak I Zhang Q Chen Y. Sensitivity and specificity of multispectral imaging for polypoidal choroidal vasculopathy. Retina. (2021) 41:1921–9. 10.1097/IAE.0000000000003130 33512897

[30] Chaikitmongkol V Kong J Khunsongkiet P Patikulsila D Sachdeva M Chavengsaksongkram P. Sensitivity and specificity of potential diagnostic features detected using fundus photography, optical coherence tomography, and fluorescein angiography for polypoidal choroidal vasculopathy. JAMA Ophthalmol. (2019) 137:661–7. 10.1001/jamaophthalmol.2019.0565 30973593 PMC6567979

[31] Yang J Yuan M Wang E Xia S Chen Y. Noninvasive multimodal imaging in diagnosing polypoidal choroidal vasculopathy. BMC Ophthalmol. (2019) 19:229. 10.1186/s12886-019-1244-5 31733642 PMC6858976

[32] Chong Teo KY Sadda SR Gemmy Cheung CM Chakravarthy U Staurenghi G Invernizzi A. Non-ICGA treatment criteria for Suboptimal Anti-VEGF Response for Polypoidal Choroidal Vasculopathy: APOIS PCV Workgroup Report 2. Ophthalmol Retina. (2021) 5:945–53. 10.1016/j.oret.2021.04.002 33866022

[33] Cheung CMG Yanagi Y Akiba M Tan A Mathur R Chan CM. Improved detection and diagnosis of polypoidal choroidal vasculopathy using a combination of optical coherence tomography and optical coherence tomography angiography. Retina. (2019) 39:1655–63. 10.1097/IAE.0000000000002228 29927796

[34] de Carlo TE Kokame GT Kaneko KN Lian R Lai JC Wee R. Sensitivity and specificity of detecting polypoidal choroidal vasculopathy with en face optical coherence tomography and optical coherence tomography angiography. Retina. (2019) 39:1343–1352. 10.1097/IAE.0000000000002139 29561386

[35] Chou YB Hsu CH Chen WS Chen SJ Hwang DK Huang YM. Deep learning and ensemble stacking technique for differentiating polypoidal choroidal vasculopathy from neovascular age-related macular degeneration. Sci Rep. (2021) 11:7130. 10.1038/s41598-021-86526-2 33785808 PMC8010118

[36] Xu Z Wang W Yang J Zhao J Ding D He F. Automated diagnoses of age-related macular degeneration and polypoidal choroidal vasculopathy using bi-modal deep convolutional neural networks. Br J Ophthalmol. (2020) 105:561–6. 10.1136/bjophthalmol-2020-315817 32499330

[37] Wongchaisuwat P Thamphithak R Jitpukdee P Wongchaisuwat N. Application of Deep Learning for automated detection of polypoidal choroidal vasculopathy in spectral domain optical coherence tomography. Transl Vis Sci Technol. (2022) 11:16. 10.1167/tvst.11.10.16 36219163 PMC9580222

[38] Cho JH Ryoo NK Cho KH Park SJ Park KH Woo SJ. Incidence rate of massive submacular hemorrhage and its risk factors in polypoidal choroidal vasculopathy. Am J Ophthalmol. (2016) 169:79–88. 10.1016/j.ajo.2016.06.014 27318076

[39] Tan ACS Jordan-Yu JM Vyas CH Gan ATL Teo KYC Chan CM. Optical coherence tomography features of polypoidal lesion closure in polypoidal choroidal vasculopathy treated with aflibercept. Retina. (2022) 42:114–22. 10.1097/IAE.0000000000003285 34412103

[40] Montorio D Giordano M Concilio M Cennamo G. Structural and vascular changes of the choroid in polypoidal choroidal vasculopathy after intravitreal anti-VEGF therapy. Ophthalmologica. (2022) 245:173–8. 10.1159/000521071 34844252

[41] Fukuyama H Komuku Y Araki T Gomi F. Association of flow signals within polyps on optical coherence tomography angiography with treatment responses after combination therapy for polypoidal choroidal vasculopathy. Retina. (2022) 42:942–8. 10.1097/IAE.0000000000003395 34954774

[42] Bo Q Zhang M Chen J Jia H Shen M Sun M. Progression of polypoidal lesions associated with exudative recurrence in polypoidal choroidal vasculopathy. Ophthalmology. (2023) 130:167–78. 10.1016/j.ophtha.2022.09.013 36152843

[43] Ma ST Huang CH Chang YC Lai TT Hsieh YT Ho TC. Clinical features and prognosis of polypoidal choroidal vasculopathy with different morphologies of branching vascular network on optical coherence tomography angiography. Sci Rep. (2021) 11:17848. 10.1038/s41598-021-97340-1 34497317 PMC8426494

[44] Azar G Vasseur V Lahoud C Favard C De Bats F Cochereau I. Polypoidal choroidal vasculopathy diagnosis and neovascular activity evaluation using optical coherence tomography angiography. Biomed Res Int. (2021) 2021:1637377. 10.1155/2021/1637377 34825001 PMC8610682

[45] Siedlecki J Klaas J Keidel L Asani B Schiefelbein J Knebel D. Optical coherence tomography-based misdiagnosis and morphological distinction in pachychoroid neovasculopathy vs. polypoidal choroidal vasculopathy. Eye (Lond). (2023). 10.1038/s41433-023-02529-5. [Epub ahead of print]. 37156864 PMC10630494

[46] Chaikitmongkol V Chaovisitsaree T Patikulsila D Kunavisarut P Phasukkijwatana N Watanachai N. Optical coherence tomography features for identifying posttreatment complete polypoidal regression in polypoidal choroidal vasculopathy. Asia Pac J Ophthalmol (Phila). (2022) 11:408–16. 10.1097/APO.0000000000000551 36179334

[47] Dat DT Hien N Quan NN Tung MQ Tam HC Hung BV. Current trends in clinical characteristics, diagnosis, and treatment of polypoidal choroidal vasculopathy: a perspective from Vietnam. J Clin Med. (2022) 11:4678. 10.3390/jcm11164678 36012915 PMC9410352

